# Comparing individual and group-level simulated neurophysiological brain connectivity using the Jansen and Rit neural mass model

**DOI:** 10.1162/netn_a_00303

**Published:** 2023-10-01

**Authors:** S. D. Kulik, L. Douw, E. van Dellen, M. D. Steenwijk, J. J. G. Geurts, C. J. Stam, A. Hillebrand, M. M. Schoonheim, P. Tewarie

**Affiliations:** Amsterdam UMC, Vrije Universiteit Amsterdam, Department of Anatomy & Neuroscience, Amsterdam Neuroscience, Amsterdam The Netherlands; Amsterdam UMC, Vrije Universiteit Amsterdam, Department of Neurology and Department of Clinical Neurophysiology and MEG Center, Amsterdam Neuroscience, Amsterdam The Netherlands; Amsterdam UMC, Vrije Universiteit Amsterdam, Brain Tumour Center Amsterdam, Amsterdam, The Netherlands; Amsterdam UMC, Vrije Universiteit Amsterdam, MS Center Amsterdam, Amsterdam, The Netherlands; University Medical Center Utrecht, Department of Psychiatry, Brain Center, Utrecht, The Netherlands

**Keywords:** Computational modeling, Magnetoencephalography, Functional connectivity, Individual prediction

## Abstract

Computational models are often used to assess how functional connectivity (FC) patterns emerge from neuronal population dynamics and anatomical brain connections. It remains unclear whether the commonly used group-averaged data can predict individual FC patterns. The Jansen and Rit neural mass model was employed, where masses were coupled using individual structural connectivity (SC). Simulated FC was correlated to individual magnetoencephalography-derived empirical FC. FC was estimated using phase-based (phase lag index (PLI), phase locking value (PLV)), and amplitude-based (amplitude envelope correlation (AEC)) metrics to analyze their goodness of fit for individual predictions. Individual FC predictions were compared against group-averaged FC predictions, and we tested whether SC of a different participant could equally well predict participants’ FC patterns. The AEC provided a better match between individually simulated and empirical FC than phase-based metrics. Correlations between simulated and empirical FC were higher using individual SC compared to group-averaged SC. Using SC from other participants resulted in similar correlations between simulated and empirical FC compared to using participants’ own SC. This work underlines the added value of FC simulations using individual instead of group-averaged SC for this particular computational model and could aid in a better understanding of mechanisms underlying individual functional network trajectories.

## Author Summary

Employing the Jansen and Rit neural mass model, we simulated individual FC by using individual SC. The AEC, one of the applied FC metrics, proved to give the highest correlations between simulated and empirical FC. Also, individual SC as compared to group-averaged SC provided higher correlations between simulated and empirical FC. However, using SC from other participants resulted in similar correlations between simulated and empirical FC compared to using participants’ own SC. Importantly, this work underlines the value of individual SC as compared to group-averaged SC to simulate FC. The insights obtained from this work might lead to a better understanding of mechanisms underlying individual functional network trajectories.

## INTRODUCTION

The brain is a complex network of brain regions that display interregional communication, that is, so-called functional connectivity (FC). FC is defined by statistical interdependencies between time series of brain activity (Friston, 2011). In case of neurophysiological data, FC can be estimated from either the phase or amplitude of neuronal oscillations (Siegel et al., 2012; Siems & Siegel, 2020). Disruption of the FC patterns are known to be clinically relevant in neurological (Stam, 2014) and psychiatric disorders (Hallett et al., 2020). Computational models are often used to gain insight into mechanisms that result in disrupted patterns of FC. Using this approach, the impact of pathology at the neuronal population level or at the level of structural connections on FC can be assessed and used to make predictions of empirical FC patterns. Especially individualized prediction of disease trajectories (Douw et al., 2019) are important in this context. However, so far mainly group-averaged structural connectivity (SC) and FC have been used, and it remains an open question whether individual predictions of FC are feasible, even in healthy conditions.

Computational modeling of brain activity and FC can be approached using so-called neural mass modeling (Deco et al., 2008). Neural mass models assume a mean ensemble activity of neurons that reduces the number of dimensions and allows multiple interacting local populations (Breakspear, 2017). A neural mass corresponds to activity within a brain region, and masses can be coupled using empirically measured structural connections, resulting in whole-brain network simulations. A well-known model that is known to generate physiologically accurate brain activity (Aburn et al., 2012) was developed by Lopes da Silva et al. (1974) and further improved by Jansen and Rit (Jansen & Rit, 1995). The Jansen and Rit model is able to produce oscillatory activity in the alpha band, that is, the dominant rhythm in resting-state neurophysiological data. Usage of this model can be justified by the fact that its dynamical properties have been thoroughly investigated and are well understood (Grimbert & Faugeras, 2006; Spiegler et al., 2011).

So far, computational modeling of empirical neurophysiological connectivity is mainly based on group-averaged SC as input to neural mass models (Abeysuriya et al., 2018; Cabral et al., 2014; Deco et al., 2017; Hadida et al., 2018; Moon et al., 2015; O’Neill et al., 2018; Tewarie et al., 2014; Tewarie et al., 2019a). One previous study on structure-function relationships compared individually simulated and empirically derived FC, based on electroencephalography (EEG) data (Finger et al., 2016). This study showed moderate to strong correlations between individually simulated and empirical FC by using a simple autoregressive model. FC was calculated with different phase-based FC metrics. Finger and colleagues tested the specificity of using individual SC by correlating individually simulated FC with either the corresponding empirical FC matrices, or with empirical FC matrices of other participants, and found no significant differences between the two approaches. This finding could be supported by a recent functional magnetic resonance imaging (MRI) study (Zimmermann et al., 2019) where it was found that the correspondence between empirical SC and FC in many participants was limited due to the small variability between participants in SC compared with the larger variability in FC, perhaps indicating that structural data is not specific enough to simulate FC accurately. Despite the relevance of previous work (Finger et al., 2016), we argue that the feasibility of individual predictions of FC should be retested in an independent dataset and should be tested using both amplitude- and phase-based metrics for FC, as recent work suggest that both phase and amplitude could encode complementary information (Siems & Siegel, 2020). However, this observation has not been reproduced in an independent dataset. In addition, we will extend previous work by including more participants, making use of magnetoencephalography (MEG) instead of EEG data and applying different FC metrics.

In the current work, we investigated how well individual empirical FC can be approximated by simulating an estimate of FC based on an individual’s own SC. We analyzed both amplitude- and phase-based metrics in this context, calculated from MEG data. To put our results into perspective, we compared our results of individual simulations with FC approximations based on group-averaged SC and individual predictions based on nonmatched empirical SC.

## METHODS

### Participants

Forty healthy participants (37.5% men, age 50.7 ± 6.1 years) from the Amsterdam multiple sclerosis cohort were included (Eijlers et al., 2018). We only included participants who underwent both diffusion MRI (dMRI) and magnetoencephalography recordings. Approval was obtained from the institutional ethics review board of the VU University Medical Center, and participants gave written, informed consent prior to participation.

### Empirical Structural Data: Diffusion MRI

Individually weighted dMRI matrices were obtained to describe the SC between the neural masses. dMRI matrices were calculated with probabilistic tractography as described previously (Meijer et al., 2020). In short, participants were scanned on a 3 T scanner (GE signa HDxt), using an eight-channel phased-array head coil. For volumetric measurements, a 3D T1-weighted inversion-prepared fast-spoiled gradient recall sequence (repetition time 7.8 ms, echo time 3 ms, inversion time 450 ms, flip angle 12°, sagittal 1.0-mm sections, 0.94 × 0.94 mm^2^ in-plane resolution) was taken into account. A diffusion-weighted imaging sequence (dMRI) was applied covering the entire brain using five volumes without directional weighting (i.e., b = 0 s/mm^2^) and 30 volumes with noncollinear diffusion gradients (echo planar imaging (EPI), b = 1,000 s/mm^2^, repetition time 13,000 ms, echo time 91 ms, flip angle 90°, 2.4-mm contiguous axial slices, 2 × 2 mm^2^ in-plane resolution). Subsequently, the FMRIB Diffusion Toolbox (part of FSL 5) was performed using eddy current distortion correction. Next, using the fiber orientation distribution (FOD), probabilistic tractography was applied using MRtrix 3.0 (Tournier et al., 2012). In this model, *N* streamlines are reconstructed by randomly putting seeds in white matter and using constrained spherical deconvolution to estimate the local FOD (Tournier et al., 2007). The 30 noncollinear diffusion directions in the data were adjusted by restricting the maximum spherical harmonic order (lmax) to 6. Then, for each participant, a random seeding of 100 million fibers within the brain mask was applied to perform whole-brain probabilistic tractography. Probabilistic tractography was applied because it is frequently used due to its low sensitivity for false positives (Maier-Hein et al., 2017).

Cortical gray matter regions were defined by processing the 3D T1-weighted image of each participant with the FreeSurfer 5.3 pipeline. The automated anatomical labeling (AAL) atlas (Tzourio-Mazoyer et al., 2002) was used to define 78 cortical regions (Gong et al., 2009) on the native cortical surface. Structural networks were constructed by considering regions as nodes and the number of fibers between pairs of nodes as links. We performed normalization of elements in the SC matrices. For each individual SC matrix, link weights that exceeded 1.5 * IQR (interquartile range) above the third quartile (Q3 + 1.5 * IQR) were set to that value, to make sure that very high values would not disproportionally influence the simulations. Subsequently, the weighted SC matrices were rescaled to the range [0 1].

### Empirical Functional Data: Magnetoencephalography

Acquisition and preprocessing of the MEG data was performed as described previously (Derks et al., 2018). In short, eyes-closed, resting-state measurements of 5 min were used. Measurements were performed in a magnetically shielded room (Vacuum Schmelze GmbH, Hanau, Germany) with a 306-channel whole-head MEG system (Elekta Neuromag Oy, Helsinki, Finland). Data were sampled at 1250 Hz, and a high-pass filter (0.1 Hz) and anti-aliasing filter (410 Hz) were employed online. The extended Signal Space Separation method (xSSS) (van Klink et al., 2017) was applied to identify malfunctioning channels. Additional malfunctioning channels were identified using visual inspection (SK, LD). We then applied the temporal extension of SSS in MaxFilter software (Elekta Neuromag Oy, version 2.2.15) on the raw data for artifact removal (Taulu & Simola, 2006). Malfunctioning channels than were identified using xSSS or visual inspection were excluded for tSSS analysis. The head position relative to the MEG sensors was recorded continuously with the signals from four or five head-localization coils. The head-localization coil positions and outline of the participants scalp were digitized using a 3D digitizer (3Space Fastrak, Polhemus, Colchester, VT, USA). Each participant’s scalp surface was coregistered to their structural MRI using a surface-matching procedure. Subsequently, the coregistered MRI was spatially normalized to a template MRI. Centroid voxels (Hillebrand et al., 2016) of the 78 cortical regions of the AAL atlas, the same as was used for the SC, were selected for further analyses after inverse transformation to the participant’s coregistered MRI. A single best fitting sphere was fitted to the outline of the scalp as obtained from the coregistered MRI and used as a volume conductor model for the beamformer approach (Hillebrand & Barnes, 2005; Hillebrand et al., 2005). An atlas-based scalar beamformer implementation (Elekta Neuromag Oy, version 2.1.28), similar to Synthetic Aperture Magnetometry (Robinson & Vrba, 1999), was applied to project MEG data from sensor level to source space (Hillebrand et al., 2012). The beamformer weights were based on the data covariance matrix and the forward solution (lead field) of a dipolar source at the voxel location. Orientation of the sources was estimated based on singular value decomposition (Sekihara et al., 2006). The broadband (0.5–48 Hz) time series of the 78 centroids were projected through the normalized (Cheyne et al., 2007) broadband beamformer weights for each target voxel (i.e., centroid voxel). From these time series, for each participant, the maximum amount of artifact free data, that is, 26 consecutive epochs of 6.55 s (8,192 samples), were analyzed (Liuzzi et al., 2017). Time series were digitally band-pass filtered in the alpha band (8–13 Hz) using a fast Fourier transform, after which all bins outside the passbands were set to zero, and an inverse Fourier transform was performed (implemented using in house script in MATLAB (version 2018b, MathWorks, Natick, MA, USA)). Subsequently, FC was calculated using different FC metrics (see section Simulated and Empirical Functional Connectivity). All the analyses in the current work were performed in MATLAB using in house scripts (see https://github.com/multinetlab-amsterdam/projects/tree/master/modelling_paper_2021).

### Simulated Functional Data: Network of Neural Masses

**Figure 1.**
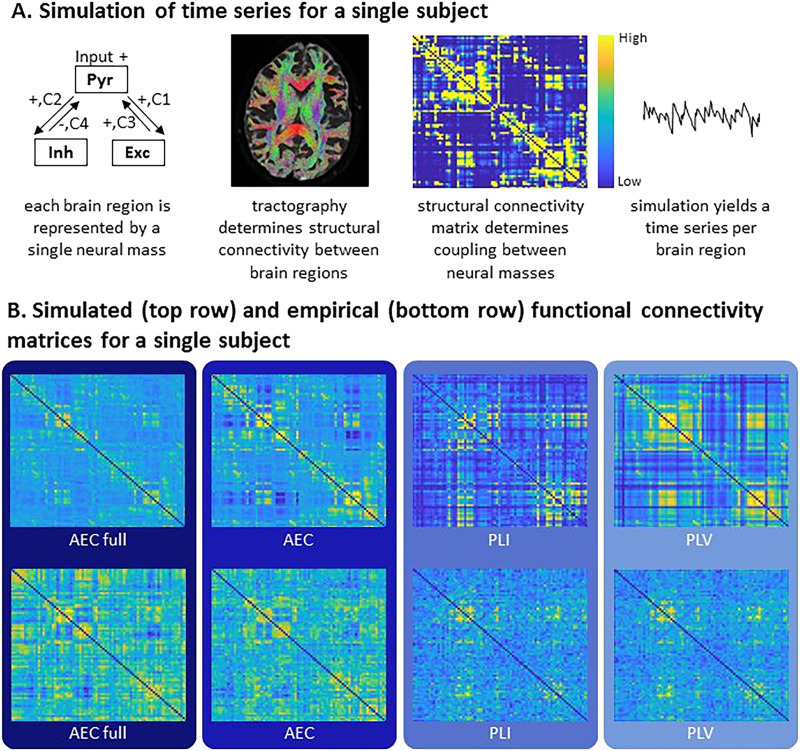
Overview of the applied methods. (A) Left: overview of the Jansen and Rit model reflecting the connections between the pyramidal (Pyr), inhibitory (Inh), and excitatory (Exc) populations. Individual weighted structural connectivity, computed by probabilistic tractography using MRTrix, was used as input to the Jansen and Rit model to connect the neural masses. Each neural mass, reflecting a brain region, produces MEG-like time series. (B) Exemplar simulated and empirical weighted functional connectivity matrices for one participant. Cold colors represent low connectivity and warmer colors represent high connectivity (this also applies to the structural connectivity matrix). For both simulated and empirical data, FC was estimated between all pairs of regions using different FC metrics. AEC = amplitude envelope correlation; AEC full = AEC calculated over the full time series (epochs concatenated), AEC, calculated over epochs; PLI = phase lag index, calculated over epochs; PLV = phase locking value, calculated over epochs. AEC full, AEC, and PLV were corrected for signal leakage in the empirical data, not in the simulated data. PLI inherently corrects for signal leakage and therefore corrects in both empirical and simulated data. For each participant and per connectivity metric, a correlation between the simulated and empirical FC was performed.

We considered a network of coupled neural masses with network size *N* = 78. Each node (neural mass) corresponded to a cortical region of the AAL atlas. Link weights (number of streamlines) were derived from an individual’s weighted SC matrix. We used the Jansen and Rit model as described in Grimbert and Faugeras (2006) to model a single neural mass. This model allows for simulation of fluctuations in the synaptic membrane potential of a neuronal population (Jansen & Rit, 1995). Each mass consists of three populations (pyramidal population, and excitatory and inhibitory neuronal populations) (see Figure 1A). The Jansen and Rit model is optimized to generate alpha oscillations. In short, each neuronal population is described by a second-order ordinary differential equation that models modulations in the mean membrane potential due to the mean incoming firing rate from the same population and from other populations in the neural mass. Incoming mean firing rates are obtained by a nonlinear sigmoid function that transforms the mean membrane potential to a mean firing rate (Jansen & Rit, 1995). Uncorrelated Gaussian noise was fed to the pyramidal population only. The three interconnected neuronal populations were connected using the coupling values (C1, C2, C3, C4) (Figure 1 and Table 1). These values represent the average number of synaptic connections between each population. Connectivity between the neural masses was implemented exactly the same as in Forrester et al. (2020), and the same parameters were used as in Grimbert and Faugeras (2006). A fourth order stochastic Runge–Kutta method (Hansen & Penland, 2006) was used to numerically solve the coupled differential equations of the model.

**Table 1.** Parameters and values included in the model (based on Grimbert & Faugeras, 2006)

**Parameter**	**Meaning**	**Value**
C1, C2, C3, C4	Average number of synapses between populations	135 * [1 0.8 0.25 0.25]
Beta_E	Time scale for excitatory population	100 ms
Beta_I	Time scale for inhibitory population	50 ms
A	Average excitatory synaptic gain	3.25
B	Average inhibitory synaptic gain	22
nu	Threshold of sigmoid	5 s^−1^
r	Slope of sigmoid	0.56 mV^−1^
theta	Amplitude of sigmoid	6 mV
Conduction velocity		10 m/s
Fs	Sample frequency	1250 Hz
h	Integration time step	0.0001
T	Observation time	20 s
P	External input to each of the neural masses	150
Coupling	Coupling between the neural masses	[0.1:0.012:0.292]

Each neural mass receives external input (P) that corresponds to external sources or activity from neighboring populations (Ableidinger et al., 2017). The external input was set to P = 150 for all neural masses. For the global coupling parameter, which determines the coupling between all neural masses, we used the interval [0.1, 0.292], with a discrete step size of 0.012. As explained in more detail later, this range was used to scan the parameter space in order to obtain the coupling value for every individual that optimized the goodness of fit between simulated and empirical FC matrices. We included distance dependent delays between nodes based on the Euclidian distance between centroids in the AAL atlas divided by the conduction velocity. See Table 1 for an overview of all model parameters. We ran the model for each global coupling value to generate time series of neuronal activity. For each run, the time series were band-pass filtered in the alpha band (8–13 Hz) in the same way as for empirical data, and FC was calculated using different FC metrics (see section Simulated and Empirical Functional Connectivity). To obtain robust results and to minimize the stochastic effect of the model’s stochastic differential equations, the model was run 20 times per subject, and subsequently FC values were averaged over the 20 runs.

### Simulated and Empirical Functional Connectivity

Three FC metrics were calculated that capture either amplitude-based connectivity or phase-based connectivity: the amplitude envelope correlation (AEC) (Brookes et al., 2011; Bruns et al., 2000; Hipp et al., 2012), the phase lag index (PLI) (Stam et al., 2007), and the phase locking value (PLV) (Lachaux et al., 1999). The AEC quantifies amplitude-based connectivity between two time series, whereas the PLI and PLV are both metrics of phase synchronization. The main difference between the latter two metrics is that the PLI inherently is insensitive to zero-lag phase differences and thereby reduces the effect of primary signal leakage. Prior to FC estimation, we first band-pass filtered the data in the alpha band (8–13 Hz) followed by correction for signal leakage. More specifically, we applied pairwise orthogonalization in order to correct for signal leakage only in empirical data and only for metrics that are inherently sensitive to signal leakage (AEC and PLV). To calculate the AEC, the amplitude envelopes were obtained from the analytical signal after a Hilbert transformation of the band-pass filtered orthogonalized time series, and the correlations between the amplitude envelopes of pairs of time series were computed. For the empirical data, the AEC was calculated in two different ways: (1) AEC: the data were divided into epochs (6.55 s), and AEC computed for every epoch. The AEC was subsequently averaged over epochs; (2) AEC full: AEC was computed for the entire time series, after concatenating all epochs. To calculate the PLI and the PLV, the instantaneous phases were obtained from the same analytical signal after the Hilbert transformation. The PLI and PLV were both calculated for every epoch (6.55 s) and subsequently averaged over epochs. For the simulated data, for each FC metric, the FC matrices were averaged over the 20 runs per coupling value.

### Similarity Between Simulated and Participant-Specific Empirical Functional Connectivity Using Individual Structural Connectivity

We computed a Spearman rank correlation (*ρ*) between simulated and empirical FC matrices for every global coupling value to quantify the match between simulated and individual empirical FC. To do this, the upper triangular part of the matrices were vectorized and subsequently correlated between simulated and empirical FC. Spearman correlations were applied since the distribution of FC values for most metrics was typically non-Gaussian. For all statistical tests performed, values of *p* < 0.05 were considered to be significant. Simulations were performed with the individual SC matrix as input to the neural mass models. The highest Spearman correlation within the coupling range [0.1, 0.292] was considered to be the best fit with the empirical FC, further referred to as the maximum correlation per participant, and calculated per FC metric. If the coupling value corresponding to the maximum correlation was at the end of the coupling range (i.e., coupling = 0.292), we extended the coupling range to 0.4, with a step size of 0.012, to test whether that coupling range would result in higher correlation values for that individual. Subsequently, the maximum correlation for the range [0.1, 0.4] was determined. Upon determining the optimal coupling per subject, the same parameters were used for all subsequent analyses.

A Wilcoxon signed rank test was subsequently performed to compare the maximum correlations between FC metrics. The FC metric that resulted in the highest maximum correlations at the group level was selected for further analyses. Differences between coupling values corresponding to the maximum correlations for the different FC metrics were tested with Friedman’s test.

### Similarity Between Simulated and Participant-Specific Empirical Functional Connectivity Using Group-Averaged Structural Connectivity

We subsequently tested whether the individual SC as input to the model outperformed simulations based on the group-averaged SC. We therefore used the average SC as input to the model and correlated the resulting simulated FC for the range of coupling values, with the individual empirical FC, using a Spearman correlation. As reference, we also predicted group-averaged FC based on simulations with the group-averaged SC as input. The group-averaged SC and FC matrices were obtained by averaging SC and FC matrices across all participants, respectively. Next, in the group-averaged weighted SC, outliers were removed and normalization of the matrix was applied as described in section Empirical Structural Data: Diffusion MRI. All subsequent steps to calculate the match between simulated and empirical FC were as described in section Similarity Between Simulated and Participant-Specific Empirical Functional Connectivity Using Individual SC.

### Simulated Versus Empirical Functional Connectivity in Matched Versus Nonmatched Participants

In a subsequent analysis, we tested whether the predictions of individual empirical FC based on participants’ own SC matrix were specific. We tested the null hypothesis that prediction of empirical FC for a given participant based on simulated FC with the SC of another participant as input to the simulations would lead to an equally well prediction. To this end, we correlated the individually simulated FC matrices, based on that participant’s optimized coupling parameter, to empirical FC matrices from other participants. We then compared the Spearman correlations between simulated and empirical FC for matched versus nonmatched data. To test whether participant’s own maximum correlation (matched data) was higher compared to the correlations obtained with all other participants’ empirical data (nonmatched data), these correlations were ranked per participant. Subsequently, if the participant’s own maximum correlation would fall within the highest 97.5% of this ranking, it was considered to be significantly higher compared to the correlations to other participants. For all previously described analyses, no corrections for multiple comparisons were performed.

## RESULTS

Exemplar time series and power spectrum of simulated data for one participant are shown in Supporting Information Figure S1. Examples of simulated and empirical FC matrices of the same participant are shown in Figure 1B.

### Similarity Between Individually Simulated and Empirical Functional Connectivity

**Figure 2.**
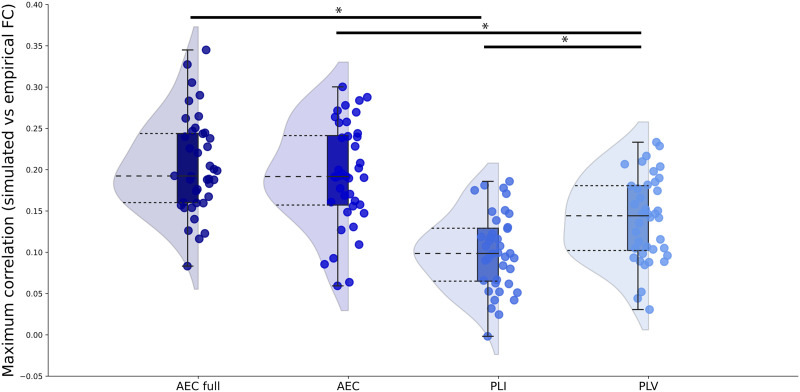
Maximum correlations between simulated and empirical FC. Rain cloud figures showing the maximum correlations between simulated and empirical FC for each FC metric. Both amplitude- and phase-based FC metrics were included: amplitude envelope correlation (AEC); AEC full refers to AEC computed over the full time series, phase lag index (PLI), phase locking value (PLV).

**Figure 3.**
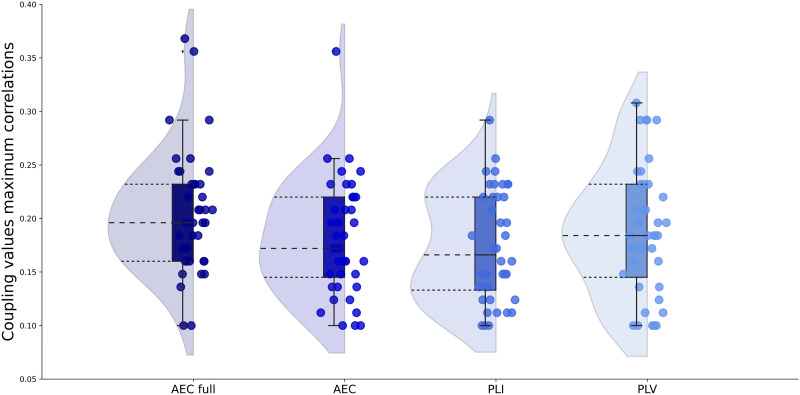
Optimal global coupling values for all FC metrics. Optimized global coupling values between neural masses as determined by the maximum correlation between simulated and empirical FC for each FC metric. AEC = amplitude envelope correlation (AEC); AEC full = AEC computed over the full time series; PLI = phase lag index; PLV = phase locking value.

The similarity between the individually simulated and individual empirical FC was calculated for each of the FC metrics for the range of coupling values. The resulting individual maximum correlation values between simulated and empirical FC are shown in Figure 2 for each FC metric. The median of the maximum correlations for each FC metric were AEC full 0.19, AEC 0.19, PLI 0.10, and PLV 0.14. All of these maximum correlations between simulated and empirical FC for the AEC full and AEC were statistically significant (for all participants with AEC full *p* < 0.001; for all participants with AEC: *p* < 0.01). For the PLI and PLV, correlations between the simulated and empirical FC were statistically significant for most participants (PLI: *p* < 0.005; PLV: *p* < 0.01), except for three (PLI) and two (PLV) participants. The coupling values corresponding to the maximum correlation between simulated and empirical FC for each participant and each FC metric are displayed in Figure 3 and Supporting Information Table S1. Coupling values corresponding to the maximum correlations did not differ between metrics (*χ*^2^ = 5.09, *p* = 0.17).

We compared individual maximum correlations between FC metrics. There was no significant difference between the AEC full and the AEC (W = 505, *p* = 0.20). AEC full showed significantly higher maximum correlations than the PLI (W = 804, *p* < 0.001), and the PLV (W = 722, *p* < 0.001). The AEC also showed significantly higher maximum correlations compared to both the PLI (W = 787, *p* < 0.001) and the PLV (W = 699, *p* < 0.001). Finally, the PLI performed significantly worse than the PLV (W = 28, *p* < 0.001) in terms of maximum correlations between simulated and empirical FC at the individual level. Since the use of the AEC full and AEC resulted in significant better predictions of individual empirical FC, we continued using only these metrics for further analyses.

Additionally, we analyzed the similarity between the strongest connections of the individually simulated and empirical data. A detailed description of this analyses can be found in the Supporting Information. For the AEC, maximal correlations between the strongest connections of simulated and empirical data showed to be significantly higher compared to the maximal correlations when the full matrices were taken into account (W = 205, *p* = 0.006, see Figure S2).

### Similarity Between Simulated Functional Connectivity and Empirical Function Connectivity Using Group-Averaged Structural Connectivity

**Figure 4.**
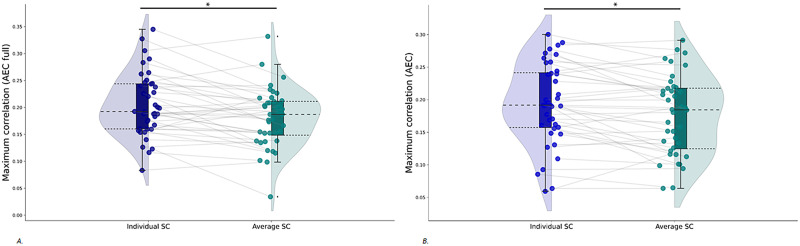
Paired rain cloud figures containing the maximum correlations, for all coupling values, between simulated and empirical FC. Gray lines between the dots connect one participant for simulations with the individual SC matrices as input to the model (blue rain clouds) and simulations with the group-averaged SC matrix as input to the model (green rain clouds). (A) FC calculated with the AEC full. (B) FC calculated with the AEC. AEC = amplitude envelope correlation (AEC); AEC full = AEC computed over the full time series.

We next predicted individual empirical FC (AEC full and AEC) based on simulations with the group-averaged SC as input. The match between simulated and individual empirical FC was better for simulations with the individual SC as input compared to simulations with the group-averaged SC as input, for both the AEC full (W = 185, *p* = 0.003) and AEC (W = 200, *p* = 0.005; see Figure 4). Results further show a median of the maximum correlations of 0.19 for both the AEC full and AEC. There was no significant difference between the maximum correlations for these two FC metrics (W = 462, *p* = 0.5).

We also computed a correlation between simulations with group-averaged SC and group-averaged FC, which showed a significant correlation between the two (AEC full: *r* = 0.40, *p* < 0.001 and AEC: *r* = 0.36, *p* < 0.001).

### Similarity Between Simulated Versus Empirical Functional Connectivity in Nonmatched Versus Matched Participants

**Figure 5.**
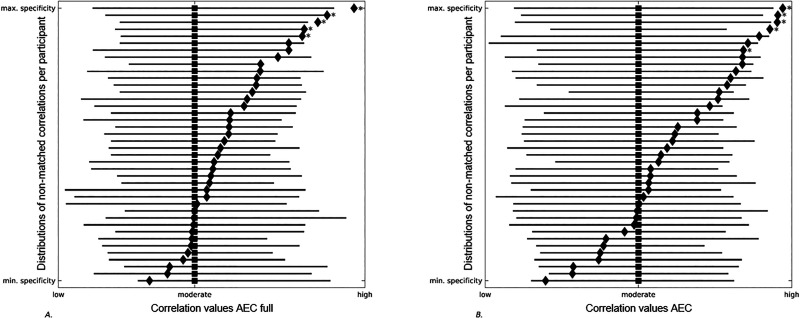
Forest plots showing the distributions of nonmatched correlations per participant. Gray lines correspond to correlation distributions (low-moderate-high) between a participants’ own simulated FC and all other participants’ empirical FC. Black squares denote median values of these distributions. Black diamonds correspond to the correlation between a participants’ own simulated and empirical FC. Gray stars display the correlations between participants’ own simulated and empirical FC that were significantly higher compared to correlations between participants’ own simulated FC and all other participants’ empirical FC. Participants are ranked based on the distance between their own correlation value and the median of all other correlation values, indicating the range between minimum and maximum specificity of participants’ own correlation values. (A) AEC full. (B) AEC. AEC = amplitude envelope correlation; AEC full = AEC computed over the full time series.

Next, we analyzed whether empirical FC of a given participant could be equally well predicted by simulated FC on the basis of another participant’s SC matrix. We correlated individually simulated FC to the empirical FC of all other participants. For both the AEC full and the AEC, in 5 out of the 40 participants, participants’ own individual correlation was significantly higher compared to the correlations with all other participants (see Figure 5).

## DISCUSSION

The main aim of this study was to assess the feasibility and accuracy of modeling individual empirical FC using individual empirical SC matrices. We found moderate correlations between simulated and empirical FC using the amplitude-based AEC, while the phase-based metrics (PLI and PLV) performed significantly worse. Using individual SC, instead of group-averaged SC, improved the correlation between simulated and individual empirical FC significantly. However, correlations between individually simulated FC and other participant’s empirical FC were in general not significantly lower than between the matched pair of FC patterns.

The FC simulations using individual SC outperformed simulations based on group-averaged SC, indicating increased precision modeling of brain activity and FC when incorporating participants’ own structural network. These findings are corroborated by Aerts and colleagues (Aerts et al., 2018), who simulated fMRI data in brain tumor patients using The Virtual Brain. Individually optimized model parameters also resulted in improved accuracy of individually simulated FC. However, when correlating an individual’s simulated FC to the empirical FC of other participants, we found correlations that were comparable to matched simulated and empirical individual FC. Although this finding is in line with earlier work (Finger et al., 2016), it remains unclear whether simulated FC can be attributed to a specific individual. It would be useful for future work to explore the causes of this apparent aspecificity. A recent study reported on subject specific MEG FC patterns, also known as functional fingerprints (Da Silva Castanheira et al., 2021). Future studies could look into such fingerprints in repeated MEG measurements over time, both between and within participants. The variation that is present between and within participants in the match between simulated and empirical FC could increase our understanding of whether the simulated or empirical FC is underlying the aspecificity that we found.

A second main result of this study is the clear difference between amplitude- and phase-based metrics in the correlations between individually simulated and empirical FC. The AEC full and AEC outperformed the PLI and PLV, while the PLV performed better in comparison to the PLI. These findings partly corroborate earlier work in which only phase-based metrics were considered (Finger et al., 2016), also showing better performance for the PLV in comparison to PLI. It is, however, important to note that Finger and colleagues used FC metrics both corrected and uncorrected for signal leakage. Although signal leakage is known to cause spurious correlations between nearby sources (Gross et al., 2013), the previously mentioned study corrected their empirical data dependent on the FC metric. Since leakage is not present in our simulated data, we therefore chose not to perform leakage correction to our simulated data, but only to the empirical data. Important to note here, however, is that the PLI inherently corrects for leakage and therefore is corrected in both our simulated and empirical data. The difference in the performance of phase- and amplitude-based metrics could relate to the consistency levels of the FC metrics. In the alpha band, the AEC has been shown to be more consistent in repeated empirical measurements from the same participants, hypothetically since phase-based metrics are more susceptible to noise (Colclough et al., 2016; Tewarie et al., 2019b). If noise indeed underlies the poorer performance of phase-based FC metrics in individual simulations, including more data, that is, including 10- instead of 5-min recordings, might improve results with these metrics (Liuzzi et al., 2017). Additionally, previous research including EEG data of patients with Alzheimer’s disease found higher reproducibility of the PLI in the theta band, while the AEC was more consistent in the alpha and beta frequency bands (Briels et al., 2020). This work could indicate that consistency of FC metrics might be frequency dependent in empirical data, an aspect that we did not take into account by only analyzing our data in the alpha frequency band.

We found moderate (*r* = 0.19 on average) correlations between individually simulated FC and individual empirical FC, which is lower than obtained by Finger and colleagues (average correlation of 0.53). However, direct comparison of these correlation values is not straightforward due to the many methodological differences between their study and ours. Nevertheless, several factors may have contributed to these results. The quality of both the empirical SC and FC matrices could have influenced the correlation strengths that we found. Regarding SC, tractography is known to underestimate the presence of interhemispheric fibers, which strongly influences modeling results (Messe et al., 2015). The tractography method we used is the current standard in the field and takes care of false positives (Maier-Hein et al., 2018). Nonetheless, future studies may investigate whether increasing the quality of the SC matrices, for instance, by improving scanner hardware, diffusion sequences, duration of scans, or the tractography methods, could enhance modeling accuracy. Furthermore, MEG data is known to be susceptible to noise caused by environmental, instrumental and biological factors. Although we only included MEG data that was visually free from artifacts, noise may still have been present in the individual FC matrices. In an additional analysis we only took the strongest connections of the simulated and empirical FC into account (Figure S2), thereby decreasing the noise of the included connections. For the AEC, the resulting match between simulated and empirical FC was higher compared to taking the full matrices into account. Furthermore, functional connections can also occur where there are few or no structural connections, possibly explained by indirect connections and interregional distance (Meier et al., 2016; Robinson, 2012). This means that even small variations in SC can support many different FC patterns, which makes the interdependence between them complicated (Popovych et al., 2019). Additionally, by correcting the empirical data for signal leakage, true zero-lag interactions are also removed, which might have been present in the simulated data, causing a decrease in agreement between simulated and empirical data.

Computational models that use average SC as an input have been frequently applied so far (Abeysuriya et al., 2018; Cabral et al., 2014; Deco et al., 2017; Hadida et al., 2018; Moon et al., 2015; O’Neill et al., 2018; Tewarie et al., 2014, 2019a), but hamper further tailoring of such models to individuals, particularly in the setting of neurological disease modeling. Previously, damage that reflects different diseases, has been modeled with advanced computational models (Aerts et al., 2020; de Haan et al., 2012; Tewarie et al., 2018; van Dellen et al., 2013), but these disease models have not yet been applied to individual data. Such tailored disease models could elucidate mechanisms underlying functional network trajectories (Douw et al., 2019) in neurological disease, for instance, modeling the impact of focal lesions on global network dysfunction and cognitive decline.

Of relevance to future computational work in health and disease, one of the limitations of our work is that we have limited our analysis to prediction of individual FC matrices and did not take into account any local aspects of the simulated data, for example, power spectral density in different brain regions. Future work using advanced and novel spectral graph based theory of neuronal oscillations could include both prediction of individual FC and first order regional power spectra (Raj et al., 2020; Verma et al., 2022). Relevant other limitations that may have played a role in the moderate fit between the simulated and empirical data in our study and the literature on how well computational modeling recapitulates empirical data relate to general confounders of MEG measurements, such as head motion, respiration, cardiac artifacts, mind wandering, and such. Although we visually inspected our data for large artifacts and used a common tool to remove such confounders from the empirical data, remaining features could have lowered the correlation with the simulated data.

To conclude, we show that simulated FC best relates to individual empirical FC when using the individual SC as input to our implementation of the Jansen-Rit model, compared to the use of group-averaged SC at least. This work therefore underlines a first step toward individual FC modeling.

## ACKNOWLEDGMENTS

The authors thank Lucas Breedt for his help with the creation of the rain cloud figures.

## SUPPORTING INFORMATION

Supporting information for this article is available at https://doi.org/10.1162/netn_a_00303.

## AUTHOR CONTRIBUTIONS

Shanna Kulik: Conceptualization; Formal analysis; Investigation; Methodology; Visualization; Writing – original draft; Writing – review & editing. L. Douw: Conceptualization; Formal analysis; Methodology; Supervision; Visualization; Writing – original draft; Writing – review & editing. E. van Dellen: Conceptualization; Methodology; Supervision; Writing – original draft; Writing – review & editing. M. D. Steenwijk: Data curation; Software; Writing – original draft; Writing – review & editing. J. J. G. Geurts: Supervision; Writing – original draft; Writing – review & editing. C. J. Stam: Writing – original draft; Writing – review & editing. Arjan Hillebrand: Data curation; Writing – original draft; Writing – review & editing. Menno Schoonheim: Conceptualization; Data curation; Formal analysis; Investigation; Methodology; Supervision; Visualization; Writing – original draft; Writing – review & editing. Prejaas Tewarie: Conceptualization; Data curation; Formal analysis; Investigation; Methodology; Software; Supervision; Visualization; Writing – original draft; Writing – review & editing.

## FUNDING INFORMATION

Menno M. Schoonheim, ZonMW Vidi grant, Award ID: 09150172010056.

## TECHNICAL TERMS

Functional connectivity: Statistical interdependencies between time series that describe activity measurements.
Computational model: Model to simulate brain activity based on empirically derived structural information, a coupling function and a local model.
Structural connectivity: Estimation of white matter tracts physically interconnect brain regions based on diffusion measurements.
Neural mass: Mean-field description of a population of neurons with usually a restricted number of state variables for each population.
MEG: Magnetoencephalography; recording the magnetic fields produced by electrical currents generated by neural populations.
dMRI: Specific MRI sequence that quantifies the diffusion of water molecules in different directions, which can be used for probabilistic tractography.
Tractography: Method for tracking the trajectory of the axonal pathways that exploits the anisotropy of the diffusion MRI signal.
